# Polymyalgia Rheumatica Originally Thought to Be Cervical Spinal Stenosis

**DOI:** 10.7759/cureus.42105

**Published:** 2023-07-18

**Authors:** Kenny Do, Eric Kawana, Jenifer Do, Ross Seibel

**Affiliations:** 1 School of Medicine, Kirk Kerkorian School of Medicine at UNLV, Las Vegas, USA; 2 School of Life Sciences, University of Nevada, Las Vegas, Las Vegas, USA; 3 Department of Pain Medicine, Optum Care, Las Vegas, USA

**Keywords:** surgery spine, shoulder and neck pain, inflammatory response, cervical spinal stenosis, polymyalgia rheumatica

## Abstract

Polymyalgia rheumatica (PMR) is an inflammatory condition that causes joint pain and stiffness. This case report describes an atypical presentation of PMR that was initially misdiagnosed as cervical spinal stenosis, leading to surgery before correctly being diagnosed with PMR. Because of an absence of specific diagnostic tests and a presentation of symptoms that often overlap with other conditions, PMR can be difficult to diagnose. This case highlights the importance of clinical evaluation and awareness of PMR’s clinical features to prevent unnecessary interventions and ensure appropriate management.

## Introduction

Polymyalgia rheumatica (PMR) is an inflammatory condition that typically presents as symmetrical rigidity and pain in joints associated with the hip, shoulder, and neck regions [1,2]. Patients who are most susceptible to this disease are usually over 50 years old, which comes second to rheumatoid arthritis in terms of prevalence for rheumatic patients in this age group [2,3]. Just like many other conditions, the incidence of PMR usually increases with age and becomes most prevalent in those who are in the seventh decade of life [4]. In fact, PMR has an incidence of 58-96 in Caucasian individuals over the age of 50 every year [1]. It is believed that minority groups are less vulnerable to the disease [1].

Although the biomedical physiology of PMR is not clearly understood, it is believed that excessive inflammation, a disordered immune system, and genetic predisposition may cause the disease [1]. High levels of C-reactive protein (CRP), elevated sedimentation rate (ESR), and IL-6 have been observed in patients who have PMR [4,5]. Abnormal white blood cells may be observed as well, including a fall in lymphocytic B, regulatory T, and TH1 cells [1,6].

In this case report, we detail an unusual case of PMR because it was originally treated as cervical spinal stenosis. The patient had uncontrolled pain in the neck and underwent spine surgery. However, the pain persisted even after the operation, which led him to consult with an interventional pain physician who diagnosed him with PMR and was able to treat his pain.

## Case presentation

A 65-year-old male patient presented to the clinic with pain in his right thoracic paraspinous parascapular region. The patient reported that he experienced axial neck pain as well. At the presentation, the patient described a deep aching and stiff sensation in those regions, where he shared that the pain ranged from 3/10 to 7/10. The symptoms are constant but are worsened when the patient performs activities that flex or extend his cervical spine, such as lifting his arms above his head. His symptoms are alleviated when he lies down with pillows supporting his back. Besides some weakness in his arms, the patient denied other complications, such as fevers, chills, weight loss, and loss of bowel or bladder functions.

During the patient interview, his medical history was unclear to the physician. The patient shared that, before seeing the current interventional pain physician, he had a series of injections to his mid-thoracic spine, and because he did not respond to treatment, he consulted with a surgeon. The patient ultimately underwent an anterior cervical disc fusion from C5-C6 and C6-C7 after the surgeon determined that he had multilevel cervical spinal stenosis. In spite of his surgery, the patient still complained of worsened pain in his neck and was not able to lift his arms above his head with stiffness to his shoulder girdles. The patient’s persistent pain led him to consult with this current interventional pain physician. Postoperative MRI imaging was unremarkable. Based on an initial evaluation of his history and symptoms, PMR was suspected.

Initially, the interventional pain physician performed a trigger point injection to the patient’s right thoracic spinous region while waiting for the completion of laboratory results. The patient’s pain did not respond to the procedure, and inflammatory markers later revealed elevated CRP (13.2 mg/L) and elevated erythrocyte sedimentation rate (38 mm/h), as seen in Table 1. Based on increased levels of inflammation, the physician consulted with the patient and started him on an oral steroid. The patient was advised to take prednisone 10 mg two times a day.

**Table 1:** Laboratory results showing increased levels of inflammation

Test	Result	Units	Flag Reference Range
ESR (Sedimentation Rate)	38	mm/h	H
C-Reactive Protein (CRP)	13.2	mg/L	H <8.0

After a one-week follow-up, the patient reported near resolution of his neck pain. He reported that he had missed work for almost a year due to his severe pain, and in response to the oral steroids, he would come back to work the following week. With the patient’s PMR improving, the interventional pain physician will recheck the patient’s inflammatory markers in one month and continue his oral steroids for a few more months. The dosage of the prednisone will be adjusted according to future laboratory results.

## Discussion

PMR can often be misdiagnosed and confused with other rheumatic diseases since there are no specific diagnostic tests for the condition [7]. In this case report, we detail a patient who was originally thought to have multilevel cervical spinal stenosis and, as a result, underwent surgery for his severe neck pain. Although the European League Against Rheumatism/American College of Rheumatology has created a provisional set of criteria that assist with diagnosing PMR, the disease is mainly identified through clinical evaluation [1,7]. Some of the criteria include patients having high CRP or ESR levels, being at or older than the age of 50, having shoulder pain in both shoulders, experiencing stiffness in the morning that lasts for over 45 minutes, and more [1]. 

PMR can mimic cervical spinal stenosis because both conditions possess overlapping symptoms, such as pain, poor motor function, and stiffness in the neck and shoulders [8,9]. Furthermore, both conditions become more prevalent in similar demographics consisting of patients over the age of 50 [8,9]. However, their etiologies are quite different. The clinical presentation of PMR involves an underlying inflammatory response to the periarticular and synovial parts of the joints [9]. Examinations of glenohumeral joints in PMR patients have shown the proliferation of major histocompatibility complex class II (MHC II) T cells and macrophages. Upregulation of vascular endothelial growth factor (VEGF) and vasoactive intestinal peptide (VIP) has been observed in PMR patients as well, both of which can enhance the mobilization of inflammatory cells [9]. In cervical spinal stenosis, however, nerves are compressed due to the narrowing of the spinal canal, which can be secondary to disc herniation, disc generation, dorsal facet hypertrophy, and trauma [10]. Unlike PMR, an inflammatory response is not seen in cervical spinal stenosis [1,8].

The inflammatory nature of PMR can be seen with it being highly associated with giant cell arteritis (GCA) [11]. In fact, GCA may arise in up to 21% of patients with PMR, where it is believed that this association may be attributed to an upregulated inflammatory response and impairment of the immune system [11]. Patients in both conditions may experience weight loss, fatigue, and muscle weakness [1]. However, patients with PMR do not usually have a high fever, a characteristic that differentiates PMR from GCA [1]. PMR has also been seen as a side effect of taking COVID-19 vaccines, with the proposed theory being an upregulation of toll-like receptors, which are proteins of the innate immune system [12].

Currently, the best treatment option for PMR is the use of low-dose oral glucocorticoids. It is recommended that patients start with the initial dose of steroids (10-20 mg) for two-four weeks, which is tapered incrementally by 2.5 mg every two-four weeks until 10 mg of steroid is achieved [13]. Afterward, the steroid is tapered by 1 mg each month until the patient's condition is completely treated [13]. 

## Conclusions

PMR is an inflammatory and immune-mediated condition that presents with pain and limited mobility to the neck and shoulders. Because of its vague symptoms, it is often confused with other rheumatic or musculoskeletal diseases. In this case report, a patient was thought to have cervical spinal stenosis due to the persistence of neck pain and stiffness. However, his symptoms did not resolve after surgery, where he was later diagnosed with PMR and was successfully treated with oral glucocorticoids. It is important for physicians to recognize PMR as an early diagnosis to effectively treat patients and reduce their pain while preventing any unnecessary operations. 
